# Cytogenetic description of the earthworm *Drawida ghilarovi* Gates, 1969 (Oligochaeta, Moniligastridae) from the southern Russian Far East

**DOI:** 10.3897/CompCytogen.v9i4.5741

**Published:** 2015-09-09

**Authors:** Alim P. Anisimov, Galina V. Roslik, Gennady N. Ganin

**Affiliations:** 1 Far Eastern Federal University, Department of Cell Biology and Genetics, 27 Oktyabrskaya str., Vladivostok 690950, Russia; 2 Institute of Biology and Soil Science, Far Eastern Branch of the Russian Academy of Sciences, 159 Av. Stoletiya Vladivostoku, Vladivostok 690022, Russia; 3 Institute of Water and Ecological Problems, Far Eastern Branch of the Russian Academy of Sciences, 65 Kim Yu Chena str., Khabarovsk 680000, Russia

**Keywords:** Karyotype, chromosomal set, nuclear DNA content, earthworm, *Drawida*, Oligochaeta, Moniligastridae

## Abstract

Sixty-six specimens of the earthworm *Drawida ghilarovi* Gates, 1969 (Oligochaeta, Moniligastridae) from 15 localities of the southern Russian Far East were studied cytogenetically. We examined chromosome sets during mitosis and diakinesis as well as DNA content in the spermatogenous and somatic cell nuclei. The populations and morphs displayed no differences in karyotype and ploidy levels estimated in terms of both chromosome number and DNA mass index: n = 10, 2n = 20; c = 1.1 pg, 2c = 2.2 pg. We conclude that polyploidy as a species- or race-forming factor is not typical of these earthworms.

## Introduction

The karyology of Oligochaeta is mainly studied in the earthworms of the family Lumbricidae. A distinctive feature of the evolution of their karyotypes is polyploidy, which is widespread in the family. The polyploid races and subspecies occur as frequently as the diploid ones (Bulatova et al. 1987, Perel 1987, Viktorov 1993, 1997, Kashmenskaya and Polyakov 2008, Garbar et al. 2009, Vlasenko et al. 2011). Di- and polyploid forms tend to be spatially isolated, with the latter occurring predominantly at the edges of the species’ ranges (Grebelnyi 2008, Kotsyuba et al. 2010, Onyschuk and Garbar 2010). In addition, conspecific races of different ploidy levels may occupy different ecological niches, vary in size and coloration, and belong to different life forms (e.g., properly edaphic and soil-litter), with *Eisenia nordenskioldi* (Eisen, 1873) from Siberia and the Russian Far East being an example (Vsevolodova-Perel and Bulatova 2008, Vsevolodova-Perel and Lejrih 2014). At the same time, it was shown that polyploidy is not the cardinal mechanism of the microevolution of the lumbricid species and subspecies in the Caucasus (Bakhtadze et al. 2008).

There are no similar data on earthworms of the family Moniligastridae, in particular, *Drawida ghilarovi* Gates, 1969. The members of this family invaded the South-Eastern Asia after the collision of the Indian and Asian lithospheric plates in the Tertiary period, i.e., 66–1.6 m.y.a. (Easton 1981). In the provinces of the North-Eastern China bordering on Russia, six tropical species of the genus *Drawida* Michaelsen, 1900 are found, and 4 and 8 *Drawida* species are reported for the Korean Peninsula and Islands of Japan, respectively (Blakemore 2003, 2007). The southern part of the *Drawida* range covers India and, possibly, Ceylon (Gates 1969, Ganin et al. 2014). *Drawida ghilarovi*, listed in the Red Books of the Russian Federation and Khabarovsk Territory, occurs in the southern Russian Far East, the northern part of its range. It was described as a new species based on its grey color morph found in forest biotopes of the Kedrovaya Pad’ and Ussuriysky Nature Reserves (Gates 1969).

Polyploidy is known to be accompanied by polymorphism. As the amount of available data increased, new color morphs of *Drawida ghilarovi* were described in the Russian literature, in particular, light-bluish (Gates 1969), aquamarine, bluish grey (Vsevolodova-Perel 1997), pitch black (Ganin 1997), greenish or bluish (The Red Book of the Russian Federation 2001), bluish-black with a metal tint, brownish, and bluish-grey (Ganin et al. 2014).

It was found out that at the northern limit the tropical moniligastridae distribution the Red Book species *Drawida ghilarovi* Gates, 1969 exists in two distinct life forms: “soil-litter” (=epigeic) inhabitants of the floodplain meadow-wetland biotopes and “aneciques” of the forest biotopes (Ganin 2013). Moreover, forest drawidas are represented by two morphs, stable in color and size, living together in different soil horizons. Larger brownish worms with a dark pigmented part close to the belt inhabit the fermentative layer A_o_; and a gray morph of worms smaller in size inhabits the 0-10 cm soil layer. Forest gray drawidas can survive in peat and wetland soils, whereas black meadow-wetland worms die in forest soils. Besides, the sympatry in the wetland and forests inhabitants is not observed, which ensures their reproductive isolation. Phenology of the two forms of worms is also different. Forest drawidas have obligate winter diapause whereas meadow-wetland species can be active all year round and do without freezing. The range of the black morph is limited to the basin of pra-Amur River in Late Neogene. Probably, the floodplain of the river had habitats typical of *Drawida ghilarovi* at that time (Ganin et al. 2014).

We are not aware of any data on the cytogenetics of *Drawida* or Moniligastridae species in general. Some information was provided in our previous report (Ganin et al. 2014). The goal of the present study is to describe the karyotype and determine the ploidy levels in geographically remote *Drawida ghilarovi* populations from various biotopes of the northern, western, southern, and eastern parts of the species’ Far Eastern range. In particular, we intended to reveal potential polyploid races or subspecies, a proposal anticipated by the presence of similar forms in lumbricids.

## Materials and methods

**Figure 1.**
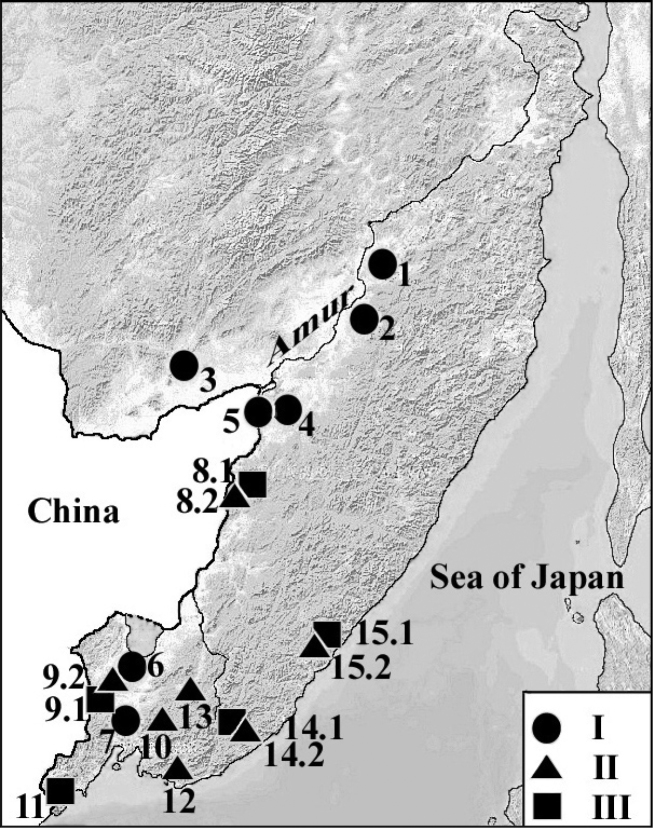
Map showing collection sites (1–15, see Table 1) of different morphs (I-III) of the tropical worm *Drawida ghilarovi* in the southern Russian Far East. I – black, black-reddish; II – brownish, yellow-brownish; III – aquamarine, bluish-grey, grey, greenish-grey, brownish-blue morphs.

Specimens of *Drawida ghilarovi* were collected in fifteen localities of the southern Russian Far East (Fig. 1, Table 1). Worms from northern part of the range were collected in the vicinity of the Slavyanka village and Anyuisky National Park (Nanaysky District, Khabarovsk Territory); and in the western part, in the Bastak Nature Reserve (Jewish Autonomous Province); in the central part of the range, in floodplain meadows of the Bolshekhekhtsirskii Nature Reserve and adjacent areas, and cedar forest in Shivki Mountain (Bikinsky District). In the Primorsky Territory, worms were sampled in submontane and montane coniferous and mixed coniferous-broad-leaved biotopes of the Ussuriysky, Kedrovaya Pad’, Sikhote-Alin’, and Lasovsky Nature Reserves, in the vicinity of Vostok Biological Station (shore of the Sea of Japan), Lazovsky Ridge (about 1000 m above sea level, Partizansky District), and in floodplain meadow biotopes of Razdol’naya River (Nadezhdinsky District) and Ilistaya River (Lake Khanka Nature Reserve, Spassky District). Based on the life form, sampled worms were subdivided into two groups: marsh-meadow and forest. Colored morphs were taken into account too. Worms were kept under laboratory conditions in accordance with soil-zoology requirements. Three sexually mature worms were taken from each of the above groups. In total 66 specimens were studied. Most of these worms were collected in summer 2010–2013 and kept under laboratory conditions.

**Table 1.** Geographical locality, life form and colored morphs of examined *Drawida ghilarovi*.

Locality No.	Locality and biotope	Geographical coordinates	Life form and colored morph
Pra-Amur and Amur Rivers basin from north to south			
1†	Khabarovsk Territory, Nanaysky District, Slavyanka village, marsh	49°27'N, 136°46'E	epigeic, black
2†	Khabarovsk Territory, Nanaysky District, Anyuisky National Park, marsh	49°20'N, 137°03'E	epigeic, black
3†	Jewish Autonomous Province, Bastak Nature Reserve, marsh	48°59'N, 135°03'E	epigeic, black
4.1†	Khabarovsk Territory, Lazo District, Bolshekhekhtsirskii Nature Reserve, floodplain of Chirki River, marsh	48°09'N, 135°08'E	epigeic, black
4.2†	The same place, marsh	48°09'N, 135°08'E	epigeic, black-reddish
5†	Khabarovsk Territory, Lazo District, Bolshekhekhtsirskii Nature Reserve, floodplain of Odyr River, marsh	48°06'N, 134°52'E	epigeic, black
6	Primorsky Territory, Spassky District, Lake Khanka Nature Reserve, meadow	44°38'N, 132°49'E	epigeic, black
7†	Primorsky Territory, Nadezhdinsky District, floodplain of Razdol’naya River, meadow	43°33'N, 131°54'E	epigeic, black
West macro-slope of the southern Sikhote-Alin			
8.1	Khabarovsk Territory, Bikinsky District, Shivki Mountain, forest	47°00'N, 134°22'E	aneciques, grey
8.2	The same place, forest	47°00'N, 134°22'E	aneciques, brownish
9.1†	Primorsky Territory, Ussuriysky Nature Reserve, forest	43°33'N, 132°21'E	aneciques, greenish-grey
9.2	The same place, forest	43°33'N, 132°21'E	aneciques, yellow-brown
10	Primorsky Territory, Mountain-taiga Biological Station, forest	43°41'N, 132°09'E	aneciques, yellow-brown
Black Mountains, Chanbaishan Plateau			
11†	Primorsky Territory, Khasansky District, Kedrovaya Pad’ Nature Reserve, forest	42°26'N, 130°38'E	aneciques, bluish-grey
East macro-slope of the southern Sikhote-Alin			
12.1†	Primorsky Territory, Vostok Biological Station, forest	42°54'N, 132°44'E	aneciques, brownish, long
12.2†	The same place, forest	42°54'N, 132°44'E	aneciques, brownish, short
13.1†	Primorsky Territory, Lazovsky Ridge, forest	43°30'N, 133°35'E	aneciques, brownish, long
13.2†	The same place, forest	43°30'N, 133°35'E	aneciques, brownish, short
14.1	Primorsky Territory, Lasovsky Nature Reserve, forest	43°00'N, 133°44'E	aneciques, grey
14.2	The same place, forest	43°00'N, 133°44'E	aneciques, brownish
15.1	Primorsky Territory, Sikhote-Alin Nature Reserve, forest	45°14'N, 136°30'E	aneciques, grey
15.2	The same place, forest	45°14'N, 136°30'E	aneciques, yellow-brown

† partly studied localities (Ganin et al. 2014), other localities are presented for the first time.

In accordance with conventional cytogenetic method, 0.04% colchicine solution was introduced into the body cavity for 18–20 h. For chromosome analysis, air-dried preparations of spermatogenous cells were made from suspended content of seminal vesicles incubated in 0.56% KCl solution and fixed with 3:1 mixture of ethanol and glacial acetic acid at 4 °C (Bulatova et al. 1987). In addition, squash preparations were made from fixed portions of seminal vesicles using an original method of tissue squashing through cellophane (Anisimov 1992). Smears of somatic cells were prepared from coelomic fluid and fixed as above. Some preparations were stained with 5% Giemsa solution, and the other, with cytochemical Feulgen nuclear reaction, which stains exclusively DNA (Bancroft and Cook 1999). Feulgen reaction allows one to use cytophotometry to measure relative DNA content in nuclei and separate chromosomes and determine cell ploidy levels. Samples of 100–150 spermatogenous cells and 30–40 coelomocytes were obtained from each worm. The cells were photographed using an AxioImager A1 and Axioscop 40 microscopes equipped with a digital camera (Carl Zeiss). The chromosome analysis and computer cytophotometry of nuclei were performed using Adobe Photoshop CS3. The relative DNA mass was obtained as follows: m(DNA) = NA*(BI – NCI), where m(DNA) is conventional DNA mass; NA, nuclear area expressed as number of pixels; BI, background intensity level; and NCI, nuclear chromatin intensity level. To estimate the absolute mass of diploid DNA (pg), cultured rat cells with known genome mass (Gregory 2005) were fixed, stained, and subject to cytophotometry under the same conditions as worm cells. For karyotypes characteristics, the length of the chromosomes was measured in micrometers at the images using AxioVision 4.8.2. Chromosomes were classified on the centromere index using the criteria of Levan et al. (1964). Centromere index is the length of the short arm divided by the total chromosome length and multiplying by 100 percentage. Data were statistically treated and histograms were built using Microsoft Excel.

## Results and discussion

Preparations made from seminal vesicles contained spermatogenous cells of different maturity depending on sampling period (May–October). In the first half of the summer, spermatogonia and lepto-, zygo-, and pachytene spermatocytes-I predominated, while in the second half, diplotene, diakinetic spermatocytes, cells undergoing meiosis, spermatids, and mature sperm cells mostly occurred. Worms collected in autumn (September–October) or spring (May) were sexually inactive. Samples obtained from them contained degenerating spermatogenous cells of different maturity and rare spermatogonia foci. In summer samples, spermatogenous cells at all maturity stages were found.

Karyotypes were determined based on the joint analysis of chromosome sets in mitotic metaphase plates of dividing spermatogonia, in spermatocytes-I at diakinesis and metaphase I of the first meiotic division. The content of nuclear DNA was measured in coelomocytes and spermatogenous cells at all maturity stages, from spermatogonia to spermatids including chromosome plates. It was also measured in spermatozoa. However, due to high optical density of chromatin, values of DNA content in spermatozoa were lower than in spermatids. They were therefore excluded from further analysis.

Photometric estimation of nuclear DNA content and chromosome analysis did not reveal genotypic differences among worms sampled from different regions or biotopes or having differences in coloration.

### Nuclear DNA content

**Figure 2.**
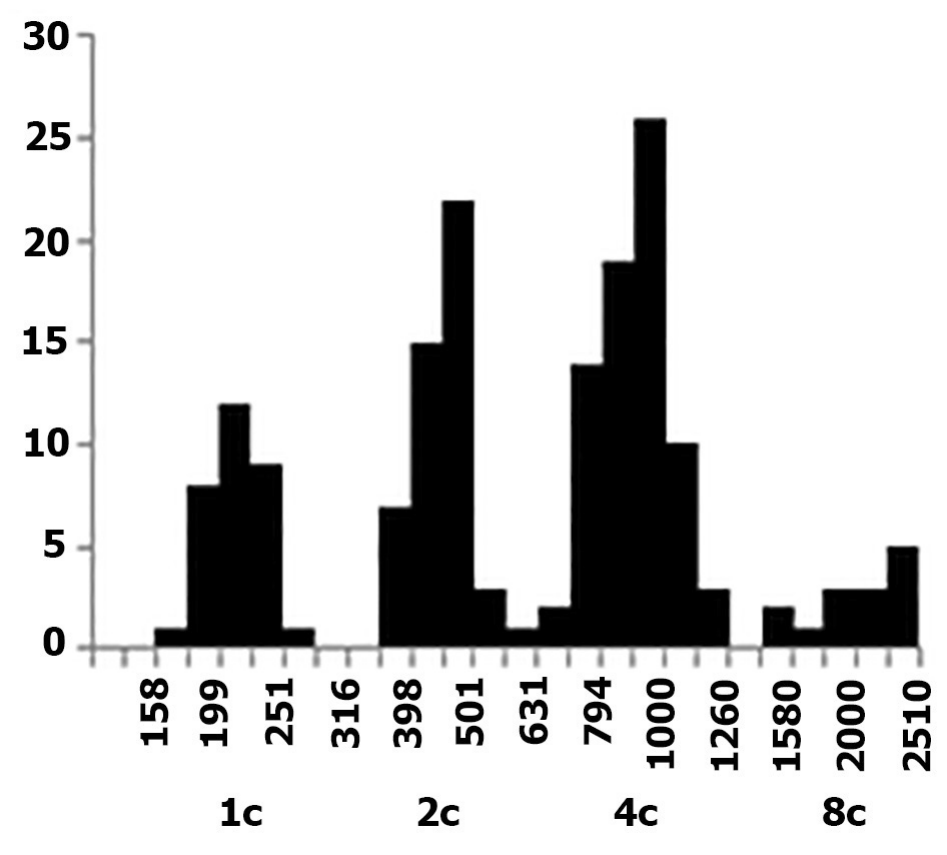
Distribution of nuclear DNA content in spermatogenous and somatic cells from seminal vesicles of *Drawida ghilarovi* sampled in the Kedrovaya Pad’ Nature Reserve. Abscissa, DNA content (conventional units and haploid “c” units). Ordinate, number of nuclei.

Nuclear DNA content in spermatogenous cells was trimodally distributed, as expected, with twofold increment between neighboring nuclear classes. In most cases, a sparse fourth class was also observed (Fig. 2). Accounting for morphological parameters of spermatogenous cells (size and shape of nuclei and chromatin structure), the class exhibiting the lowest DNA content was attributed to spermatids and spermatozoa; it is therefore a haploid class (c). The next class, a diploid one (2c), was formed by spermatogonia, rare spermatocytes II, and somatic cells of seminal vesicles. The tetraploid class (4c) comprised premeiotic spermatocytes-I (including easily identifiable pachytene, diplotene, and diakinetic stages) and mitotic and G_2_-phase spermatogonia. The small octoploid class (8c), atypical of sexual cell population, was formed by apparently meiotic prophase nuclei twice as large as normal tetraploid nuclei.

**Figure 3.**
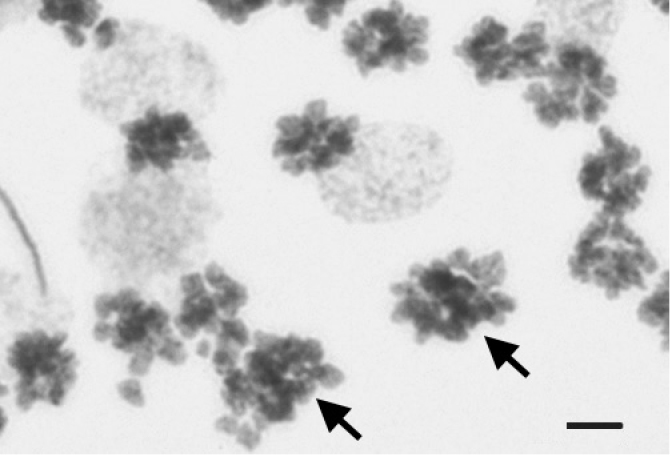
Cluster of synchronously dividing spermatogonia from a seminal vesicle of *Drawida ghilarovi* sampled in the Lasovsky Nature Reserve. Arrows point at two tetraploid (4n8c) mitoses among ordinary diploid ones (2n4c). Scale bar: 5 µm.

In accordance with these results, tetraploid (4n8c) mitotic figures occurred in clusters of normal diploid mitoses (Fig. 3). Obviously, a small portion (<0.5–1%) of spermatogonia undergo polyploidizing mitosis in the last cell cycle to form a subpopulation of anomalous spermatocytes with octoploid DNA content. In several cases, diakinetic nuclei with an increased number of bivalent chromosomes were encountered. Diploid spermatids and spermatozoa resulting from such polyploid meiosis only occasionally occurred. Anyway, their number did not correspond to that of divided octoploid spermatocytes.

The estimates of DNA content in coelomic fluid cells confirm the above results, in particular, the presence of octoploid spermatocytes. Coelomocytes used as a coarse reference of diploid DNA content normally had the expected DNA amount (2c), coinciding with the second peak of the distribution of DNA content in sexual cells (appr. 450 conventional units). Occasionally, the distribution of DNA content in coelomocytes displayed hypodiploid asymmetry, possibly, due to mass degradation (apoptosis) of these cells.

Polyploidization of a portion of spermatogenous cells may be considered analogous to somatic polyploidy (endopolyploidy, localized polyploidy) widespread in plants and animals. In somatic polyploidization, a portion of a cell population (occasionally, the whole population) switches to incomplete mitotic cycles including abortive mitosis, endomitosis, or DNA endoreplication in polytene chromosomes (Edgar and Orr-Weaver 2001, Anisimov 2005, Lee et al. 2009, Davoli and Lange 2011). In *Drawida* spermatogenous cells, the polyploidy is facultative and has no apparent adaptive or population-genetic value.

Using cytophotometry of spermatogenous cells and coelomocytes, the averaged diploid DNA content (in conventional units) was estimated for each of *Drawida ghilarovi* populations (life forms and colored morphs) (Table 2). As is seen, different populations displayed no substantial differences in 2c DNA content of the standard (diploid) chromosome set. The estimates varied from 433±6 to 479±8 conventional units. Some between-population differences in 2c DNA content were statistically significant. For example, the values 442±9 (locality No. 1) and 463±5 (No. 3) differ with p<0.05; 462±7 (No. 9.1) and 436±5 (No. 9.2) display p<0.01; while limits 433±6 (No. 8.1) and 479±8 (No. 10) have p<0.001. Noteworthy, similar differences were occasionally observed between samples from the same population. Probably, they are the result of methodical or seasonal variations during collection and/or keeping of worms. Anyway, the present results revealed no signs of organism-level polyploidy in the *Drawida ghilarovi* populations studied, which would cause genome-scale differences as early as in zygote.

**Table 2.** Mean diploid DNA content in *Drawida ghilarovi* locality as determined by cytophotometry. SE – standard error.

Locality No. (see table 1)	Mean 2c DNA content ± SE (in conventional units)	Locality No. (see table 1)	Mean 2c DNA content ± SE (in conventional units)
1†	442±9	9.2	436±5
2†	458±9	10	479±8
3†	463±5	11†	477±5
4.1†	444±8	12.1†	445±8
4.2†	465±9	12.2†	442±6
5†	467±8	13.1†	448±8
6	474±6	13.2†	460±8
7†	467±9	14.1	468±7
8.1	433±6	14.2	467±7
8.2	442±6	15.1	466±8
9.1†	462±7	15.2	470±5

† partly studied localities (Ganin et al. 2014), other localities are presented for the first time.

The size of *Drawida ghilarovi* genome expressed as absolute DNA mass (pg) was estimated as follows. The photometric amount of 2c DNA averaged for 22 samples was 458 conventional units. Hence, c = 229 conventional units. The photometric amount of rat 2c DNA determined from cultured cell preparations using the same staining protocol was 1284 conventional units, which gives 642 conventional units per haploid amount (c). The absolute haploid DNA mass of the rat genome is 3.1 pg (see reference base in Gregory 2005). The proportion (229 × 3.1 : 642) gives the haploid (c) mass of *Drawida ghilarovi* DNA to be 1.1 pg.

### Karyotype characteristics

**Figure 4.**
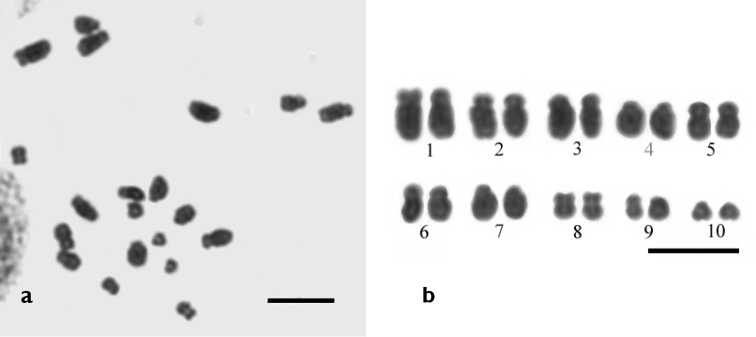
Mitotic metaphase (**a**) and karyogram (**b**) of *Drawida ghilarovi* from the Sikhote-Alin Nature Reserve. 2n = 20. Scale bars: 5 µm.

Chromosome analysis revealed that worms belonging to different populations and color morphs, with dividing spermatogonia having 20 chromosomes in the diploid set (2n = 20) (Fig. 4a). The pairing of homologous chromosomes is easily discerned in karyogram (Fig. 4b). As is seen, the *Drawida ghilarovi* karyotype comprises 10 chromosome pairs, whose mean length, centromeric index and morphology are presented in the Table 3.

**Table 3.** Mean length (ML), its standard deviation (SD) and centromere index (CI) of the chromosome pairs in six metaphase plates of *Drawida ghilarovi*. SE – standard error; m – metacentric, sm – submetacentric, st – subtelocentric chromosomes.

Chromosome pair	ML ± SE (µm)	SD of ML	CI ± SE	Centromere position
1	3.03±0.05	0.17	28.97 ±1.18	sm
2	2.71±0.07	0.25	27.65±1.14	sm
3	2.46±0.05	0.16	29.91±1.47	sm
4	2.33±0.06	0.21	24.10±0.37	st
5	2.20±0.05	0.17	44.93±0.87	m
6	2.06±0.06	0.19	30.16±1.62	sm
7	1.94±0.05	0.15	22.92±0.38	st
8	1.75±0.04	0.13	38.91±0.82	m
9	1.53±0.04	0.13	39.80±0.97	m
10	1.23±0.05	0.16	23.12±0.62	st

**Figure 5.**
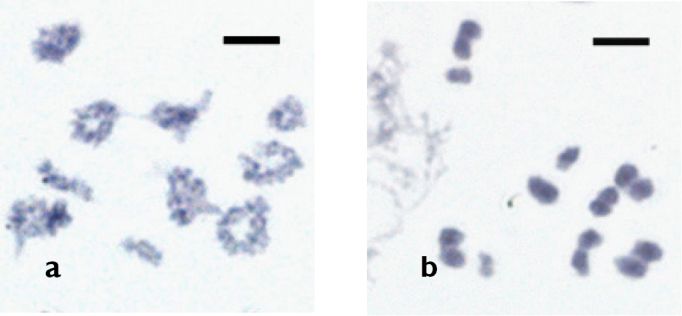
Early diakinesis (**a**) and meiotic metaphase-I (**b**) in *Drawida ghilarovi* from the Kedrovaya Pad’ Nature Reserve. n = 10. Scale bars: 5 µm.

At early diakinesis almost all bivalents had a ring-like morphology, except two bivalents which were rod-shaped (Fig. 5a). The analysis of spermatocytes-I at the stages of diakinesis to metaphase-I containing the haploid number of bivalent chromosomes also showed that all *Drawida ghilarovi* specimens had the same chromosome set (n = 10) (Fig. 5). In accordance to this, 10 single chromocenters could be observed in early spermatids.

The differences between chromosomes in relative DNA content were determined by cytophotometry of separate bivalents in several diakinetic plates. Their ranked series is presented in Table 4. It begins with bivalent 1, the largest one (15.8% of the total DNA content) and ends with bivalent 10 only comprising 3.6% of the total DNA content. In further research, these data together with morphometric results may be used as an additional parameter when comparing *Drawida* karyotypes from new ranges.

**Table 4.** DNA cytophotometry data of genome mass (%) distribution in chromosomes of individual diakinetic spermatocytes of *Drawida ghilarovi*. M – mean, SE – standard error.

Locality No. / specimen No.	Chromosome No.									
	1	2	3	4	5	6	7	8	9	10
1/1	16.01	14.07	13.12	10.83	10.75	10.00	9.87	6.63	5.27	3.46
3/1	15.70	14.27	12.95	10.50	10.17	10.12	9.51	6.54	6.54	3.71
6/1	15.84	13.80	11.95	11.17	11.25	9.89	9.88	6.74	6.06	3.41
6/2	15.68	13.47	12.43	10.93	10.15	9.92	9.61	7.96	6.16	3.69
7/1	15.30	13.48	12.85	11.07	10.88	9.93	9.40	6.90	6.38	3.80
11/1	16.36	14.05	12.76	11.62	11.43	10.29	9.35	5.74	4.80	3.60
11/2	15.40	12.94	12.93	11.58	11.38	10.25	9.97	6.21	5.85	3.48
M ± SE	15.76 ±0.14	13.73 ±0.17	12.71 ±0.15	11.10 ±0.15	10.86 ±0.20	10.06 ±0.06	9.65 ±0.09	6.67 ±0.26	5.87 ±0.24	3.59 ±0.06

As was mentioned above, there are no available data on the cytogenetics of Moniligastridae, in particular, *Drawida*. However, karyotypes of Lumbricidae worms are relatively well studied. Approximately half of the members of this group are polyploid. In most lumbricids, the basic haploid set includes 18 chromosomes, and diploid, 36 chromosomes (Viktorov 1993, Vsevolodova-Perel and Bulatova 2008, Bakhtadze et al. 2008, Kashmenskaya and Polyakov 2008). In polyploid members of the family, the number of chromosomes is usually a multiple of 18. However, some diploid species of the family have haploid sets of 11, 15, 17, or 19 chromosomes (Bakhtadze et al. 2008, Onyschuk and Garbar 2010). For example, in different populations of *Eisenia foetida*, the haploid and diploid sets comprise 11 and 22 chromosomes, respectively (Vitturi et al. 1991, Viktorov 1993, Bakhtadze et al. 2008). Thus, *Drawida ghilarovi* has the smallest chromosome set among all earthworms studied so far (n = 10 and 2n = 20). In the family Lumbricidae, the chromosome size varies usually within 2–10 µm, while the largest chromosome in *Drawida ghilarovi*, scarcely exceeds 3 µm (cf. Fig. 4 and Table 3), and the size of incompletely compacted chromosomes in that, up to 5 µm (Ganin et al. 2014). At the same time, the genome mass in *Drawida ghilarovi* presently estimated to be about 2.2 pg (2c) is substantially greater than in other lumbricids. Thus, in *Octodrilus complanatus*, 2c = 1.72 pg (at 2n = 36), and in *Eisenia foetida*, 2c = 1.4 pg (at 2n = 22) (Vitturi et al. 2000). Obviously, comparative cytogenetic characterization of Oligochaeta requires further research using various morphological and cytochemical parameters.

## Conclusion

To summarize the above, all examined *Drawida ghilarovi* populations from the southern Russian Far East had the same karyotype and ploidy level in terms of both chromosome number and DNA mass, exactly, n = 10, 2n = 20; c = 1.1 pg, 2c = 2.2 pg. In other words, polyploidization as a species- or race-forming factor is not typical of this group.
